# Tamm–Horsfall Protein is a Potent Immunomodulatory Molecule and a Disease Biomarker in the Urinary System

**DOI:** 10.3390/molecules23010200

**Published:** 2018-01-22

**Authors:** Tsai-Hung Wu, Ko-Jen Li, Chia-Li Yu, Chang-Youh Tsai

**Affiliations:** 1 Division of Nephrology, Taipei Veterans General Hospital and National Yang-Ming University, Taipei 112, Taiwan; thwu@vghtpe.gov.tw; 2 Division of Rheumatology, Immunology & Allergy, Department of Internal Medicine, National Taiwan University Hospital, Taipei 100, Taiwan; dtmed170@gmail.com (K.-J.L.); chialiyu@ntu.edu.tw (C.-L.Y.); 3 Division of Allergy, Immunology & Rheumatology, Taipei Veterans General Hospital and National Yang-Ming University, 201 Shih-Pai Road, Sec 2, Taipei 112, Taiwan

**Keywords:** Tamm–Horsfall protein (THP), immunomodulation, renal tubular biomarker, polymorphonuclear leukocyte phagocytosis, protein-binding capacity

## Abstract

Tamm–Horsfall protein (THP), or uromodulin (UMOD), is an 80–90-kDa phosphatidylinositol-anchored glycoprotein produced exclusively by the renal tubular cells in the thick ascending limb of the loop of Henle. Physiologically, THP is implicated in renal countercurrent gradient formation, sodium homeostasis, blood pressure regulation, and a defense molecule against infections in the urinary system. Investigations have also revealed that THP is an effective binding ligand for serum albumin, immunoglobulin G light chains, complement components C1 and C1q, interleukin (IL)-1β, IL-6, IL-8, tumor necrosis factor (TNF)-α, and interferon-γ through its carbohydrate side chains for maintaining circulatory and renal immune homeostasis. Thus, THP can be regarded as part of the innate immune system. *UMOD* mutations play crucial roles in congenital urolithiasis, hereditary hyperuricemia/gout, and medullary cystic kidney diseases. Recent investigations have focused on the immunomodulatory effects of THP on immune cells and on THP as a disease biomarker of acute and chronic kidney diseases. Our studies have suggested that normal urinary THP, through its epidermal growth factor (EGF)-like domains, binds to the surface-expressed EGF-like receptors, cathepsin G, or lactoferrin to enhance polymorphonuclear leukocyte phagocytosis, proinflammatory cytokine production by monocytes/macrophages, and lymphocyte proliferation by activating the *Rho* family and mitogen-activated protein kinase signaling pathways. Furthermore, our data support both an intact protein core structure and carbohydrate side chains are important for the different protein-binding capacities of THP. Prospectively, parts of the whole THP molecule may be used for anti-TNF-α therapy in inflammatory diseases, autoantibody-depleting therapy in autoimmune disorders, and immune intensification in immunocompromised hosts.

## 1. Introduction

**Figure 1:**
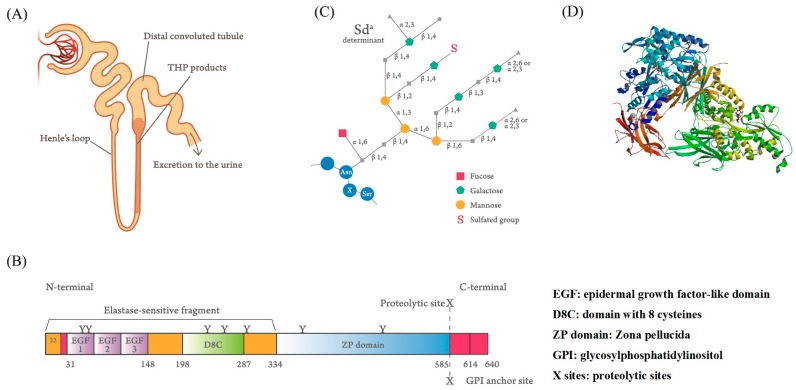
Synthesis site, protein domain structure, and fine tetra-antennary carbohydrate compositions of normal human Tamm–Horsfall protein (THP). (**A**) The thick ascending limbs of the loop of Henle in the nephrons are the essential synthesis sites of THP. The molecule is directly excreted into the urine stream after production. (**B**) The domain structure of the THP molecule shows 3 epidermal growth factor (EGF)-like domains (marked by EGF-1, EGF-2, EGF-3), 7 *N*-glycosylation sites (marked by Y), and a proteinase cleavage site (marked by X) (adapted from Li et al. [36]). (**C**) Fine tetra-antennary carbohydrate composition of THP and its binding site with the blood substance Sd^a^ (from Serafini-Cessi et al. [37]). (**D**) 3-D structure of THP (from Bokhove et al. [38]).The full names of the abbreviations in the scheme are as follows: α1,3: Siaα(1,3)-galactose/*N*-acetylgalactosamine; α1,6: Siaα(1,6)-galactose/*N*-acetylgalactosamine; α2,3: Siaα(2,3)-galactose/*N*-acetylgalactosamine; α2,6: Siaα(2,6)-galactose/*N*-acetylgalactosamine; β1,2: β(1,2)-*N*-acetyl-glucosamine oligomers; β1,3: β(1,3)-*N*-acetylglucosamine oligomers; β1,4: β(1,4)-*N*-acetylglucosamine oligomers; β1,6: β(1,6)-*N*-acetylglucosamine oligomers.

Igor Tamm and Frank Horsfall [1,2,3] first purified a mucin-like glycoprotein that could inhibit virus-induced hemagglutination in vitro and bears their name from normal human urine. It was also found that Tamm–Horsfall protein (THP or uromodulin, UMOD) acted as a crucial defense molecule against viral and bacterial infections in the urinary tract by its binding activity [4,5,6]. The synthesis site of this 80–90-kDa carbohydrate (CHO)-rich glycoprotein is identified exclusively in the renal thick ascending limb (TAL) of the loop of Henle [7,8]. The side chains of the THP molecule are constituted by approximately 25–30% of CHO moieties [9,10,11,12]. This intricate glycosylation structure not only allows THP aggregation at different pH levels and high salt concentrations, dissolution in alkaline pH, and binding with lectin-like molecules on microorganisms and serum proteins, but also exerts immunomodulation on immune-related cells [13,14,15,16,17,18,19,20,21,22,23,24,25,26]. Recently, Micanovic et al. [27] found that THP could negatively regulate bone marrow granulopoiesis and suppress neutrophil infiltration in the damaged kidney through inhibition on the renal epithelial interleukin (IL)-23/IL-17 axis. *UMOD* mutations result in different congenital disorders including progressive distal tubular dysfunction [28,29], familial juvenile hyperuricemic nephropathy [30], urinary tract stone formation [31,32], salt-sensitive hypertension, and kidney damage [33,34]. The synthesis site (Figure 1A), domain structure (Figure 1B), and fine tetra-antennary CHO side chains of THP (Figure 1C) are illustrated in Figure 1, which demonstrates that THP contains a florid CHO side chain structure, mainly involving *N*-linked glycans of di-, tri-, and tetra-antennary branches and an *N*-glycosylation site with high-mannose sequences [10,11,12]. The molecule is composed of 3 epidermal growth factor (EGF)-like domains (marked by EGF-1, EGF-2, and EGF-3), 7 *N*-glycosylation sites (marked by Y), and a proteinase cleavage site (marked by X). Once produced, the molecule is directly excreted into the urine stream but not into the renal interstitium or blood. In healthy individuals, the daily urinary THP excretion ranges from 50 to 150 mg to protect hosts from pathogenic microbial invasions by compensating the low production or absence of antibodies, complements, or immunocompetent cells in the urinary system [35].

In this review, we discuss in detail the classic and novel physiological functions, unique immunological/immunomodulatory functions, and pathological conditions related to decreased synthesis, aberrant localization, and *UMOD* mutation, as well as future prospective clinical applications of THP.

## 2. Physiological Functions of THP

### 2.1. Classic Physiological Functions of TAL Relevant to Chemical and Biochemical Properties of THP

THP is a highly glycosylated peptide with a high molecular weight up to 1 million Da in aggregate that can be dissociated into monomeric subunits (molecular weight, 95 kDa) by guanidine, acetic acid, urea, or sodium dodecyl sulfate [39]. The molecule contains high amounts of cysteine in the range of 1 per 11–12 amino acid residues [10,11,12,40,41]. Moreover, the acidic amino acids outnumber the basic ones, giving THP a low isoelectric point and poor dissolution properties in neutral solution or water. Once dissolved in alkaline solution, it tends to exist in a gel-like form. This physical property renders THP an essential component of urinary hyaline casts [42]. However, when the glycosylation process is disturbed, its cast formation capacity may be lost. Similar to other glycoproteins, the CHO content of THP varies considerably, reaching as high as 30% [40,41] in normal urine or considerably higher in urine during pregnancy [14]. With immunohistochemical staining, THP can be detected only in the distal part of the nephron, particularly in the cells of the TAL of the loop of Henle, where active water and chloride reabsorption occur [43,44,45]. Thus, THP is not only a useful antigenic biomarker for examining the renal tubule but also plays an important role in the modulation of normal TAL function. Physiologically, a large amount of NaCl in the glomerular filtrate is actively reabsorbed in the TAL [43]. Because the permeability of the TAL is extremely low compared with that of the proximal tubules, the tubular fluid generated and delivered to the distal convoluted tubule is hypotonic to plasma, thus resulting in medullary countercurrent gradients [44]. The unique electrolyte-dependent reversibly aggregating feature of THP may lead to microgel formation and increased viscosity in the TAL, where the electrolyte concentration is high [46]. Indeed, active chloride transport and passive movement of cations into the lateral intercellular spaces would create an ionic environment favorable for the aggregation of THP present on the lateral intercellular membrane. Previous investigators have postulated that THP may act as the exact 2Cl^−^-K^+^-Na^+^ cotransporter. However, immunohistochemical staining of a variety of renal epithelia failed to colocalize THP with the distribution of the 2Cl^−^-K^+^-Na^+^ cotransporter [45]. 

### 2.2. Novel Physiological Functions of THP

#### 2.2.1. Physiologic Processes of THP Relevant to Its Lectin-binding Activity 

**Figure 2:**
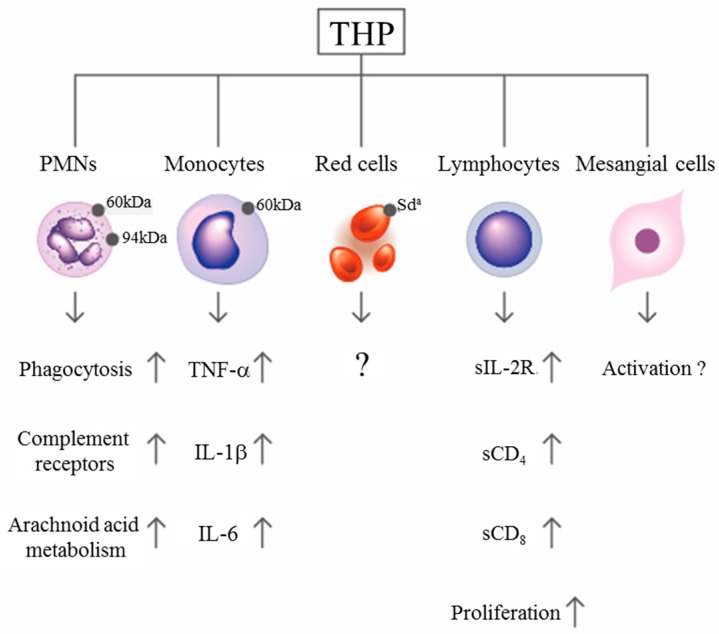
Scheme showing the immunological effects of Tamm–Horsfall protein (THP) after binding with surface receptors on different blood cells.

THP is a crucial defense molecule in the urinary system against infections, intrinsic offending molecules, and damaged kidney-induced inflammation. The THP molecule, with its intricate CHO side chain structures, binds not only with exogenous microbial pathogens [1,2,3,4,5,6] but also with harmful intrinsic offending molecules such as the blood group antigen Sd^a^ [47] (Figure 2), extracellular matrix [48], and lectin-like molecules [49,50]. THP is insoluble in an acid milieu and, with its low isoelectric point, can readily aggregate into jelly-like form and become a urinary cast. Scanning electron microscopy revealed that THP showed a filamentous structure with a diameter of 15–45 nm and an alternatively intermingled topography constituting a 3-dimensional meshwork with sub-micrometer pores [51]. This “trapping net”-like structure is essential for ensnaring microorganisms and facilitating their elimination from the urinary tract by voiding. Bates et al. [52] demonstrated that ablation of *UMOD* increased the susceptibility of mice to bacterial colonization in the bladder, type 1-fimbriated *Escherichia coli* infection, and ureteral stone formation. Clinically, decreases in THP production by the kidneys in diabetic nephropathy, lupus nephritis, or end-stage renal disease may lead to susceptibility to repeated urinary tract infections [53,54]. In addition, a decrease in urine THP excretion can serve as a biomarker for predicting the onset of renal tubular damage from different etiologies [53,54,55,56,57,58]. Notably, THP exerts a protective effect from active kidney injury by inhibiting neutrophil infiltration [27]. Heitmeier et al. [57] demonstrated that tumor necrosis factor (TNF)-α can activate hepatocyte nuclear factor-1 to regulate THP expression in the TAL of the loop of Henle during kidney injury for further protection from tissue inflammation.

#### 2.2.2. THP is a Major Regulator of Systemic and Renal Immune Factor Homeostasis

THP has been reported to play a housekeeping role in regulating systemic and renal immune factor homeostasis [13,59]. Function–structure relationship studies have shown that the tetra-antennary CHO residues of THP play an important role in this protein’s lectin-binding affinity with the proinflammatory cytokine IL-1β, IL-2, or TNF-α and may act as a cytokine trap to regulate circulating and renal immune homeostasis [13,17,18,19]. Furthermore, the specific *O*-linked chains exert potential ion interactions with various serum proteins including immunoglobulin G (IgG) light chains [20] as well as complement components C1 and C1q [15,16]. However, the CHO side chains of THP and the hydrogen ion concentration in an interstitial milieu are important for its interactions with complement C1q and inhibition in the classical complement activation pathway [15,60]. Wu et al. [61] indicated that the intact protein core structure of THP is essential for this protein’s binding with other proteins and immune cells. The same group measured the binding affinity (*K*_d_) between THP and TNF-α by Scatchard plot analysis and revealed that the binding affinity of THP and TNF-α was 1.4–1.7 × 10^−6^ M, which is lower than the antigen–antibody or ligand–receptor binding affinity [62]. They also identified that β(1,4)-*N*-acetylglucosamine oligomer (GluNAc) and GluNAc/branched mannose in bovine serum albumin, IgG, and TNF-α are essential for binding with THP. Moonen et al. [63] also attempted to study THP–cytokine binding in solution. They found that THP interacts with denatured TNF-α but not native, soluble TNF-α at a pH below 6.0, which is an acidic milieu likely to be encountered in the distal convoluted tubules in vivo. They argued that investigations using in vitro immobilized cytokines in solid phase as a capture reagent could not reflect the in vivo condition of the active cytokines. However, immunohistochemical staining has demonstrated that exogenously administered, ^125^I-labeled recombinant IL-1, IL-2, and TNF-α were rapidly cleared from the circulation with an average half-life of only 5–10 min and were found to bind to renal tubular segments that express THP in vivo [64,65,66]. These experiments suggest that the binding of cytokines with THP in renal tubules is not dependent merely on a low-pH milieu. The high-salt environment in the loop of Henle may also further denature the circulating cytokines to facilitate binding with THP. 

## 3. Immunological Functions of THP

### 3.1. Immunomodulatory Activities of THP

By using a concanavalin A adherence column, Muchmore and Decker [67] found an 85-kDa immunosuppressive glycoprotein in the urine of pregnant women with a gestational period longer than 20 weeks. They named it uromodulin, but it was subsequently confirmed by Pennica et al. [68] to share the same peptide backbone as THP. The same molecule was also found in human amniotic fluid in considerable amounts [69], indicating that THP/UMOD is important in protecting a fetus against anti-alloantigen antibody attack and preventing T-cell-mediated allogeneic rejection by a mother who confronts a half-difference of the haplotype genes in her baby. Structural assessment revealed that the glycosylation of THP molecules undergoes considerable modifications in some situations such as pregnancy [14], diabetes mellitus [70], and renal allograft rejection [71]. THP can also adhere to the surface of various blood cells including erythrocytes (RBC), polymorphonuclear leukocytes (PMN), lymphocytes, monocytes/macrophages/dendritic cells, and glomerular mesangial cells [21,22,23,24,25] to enhance PMN phagocytosis [21,22,25], lymphocyte proliferation [21,24,72], and mononuclear phagocyte activation [73]. More recently, THP present in renal interstitium was found to positively regulate mononuclear phagocyte number, plasticity, and phagocytic activity, which might play essential protective role during acute renal injury [74]. The binding and activation effects of THP on RBC and different immunocompetent cells are summarized in Figure 2. Previously, we observed that the molecular basis of THP-induced, monocyte-dependent lymphocyte proliferation might implicate THP binding, membrane depolarization, and increased sodium uptake, as well as increased expression of IL-2R and major histocompatibility complex class II antigens on the cell surface [72]. Furthermore, overnight incubation of THP with peripheral blood mononuclear cells (PBMCs) facilitates the synthesis of IL-1β, IL-6, TNF-α, gelatinase, and superoxide anion radicals in a dose-dependent manner [23,72,73]. In addition to these immune-stimulating activities, many authors have noted the inhibitory activities of THP on T-cell responses. Su et al. [24] observed the dynamic responses of pro- and anti-inflammatory cytokine production from human mononuclear cells induced by THP. Franceshi et al. [75] showed that THP per se could inhibit alloantigen-induced lymphocyte blastogenesis in one-way mixed lymphocyte reactions. Winkelstein et al. [76] demonstrated that THP is a specific inhibitor of IL-1-initiated human T-cell colony formation. These results have suggested that THP may exert immunosuppressive effects on lectin-, antigen-, or allograft-induced lymphocyte proliferation in addition to immune potentiation on naïve immune cells [26,66,75,76]. The surface receptors on immune-related cells responsible for THP binding have also been elucidated by some authors. Saemann et al. [77] reported that THP links innate immune cell activation with adaptive immune responses through Toll-like receptor 4. Pfistershammer et al. [78] identified the scavenger receptors SREC-I, CLA-1 (SR-B1), and SR-A1 as cellular receptors for THP. However, Siao et al. [79] found that the surface-expressed lactoferrin and cathepsin G on human PMN could serve as receptors for THP binding. After binding with surface receptors on naïve immune cells, THP may stimulate different cytokine production patterns to activate lymphocyte proliferation (Figure 2). Notably, THP may conversely exert inhibitory activity on activated mononuclear cells to suppress cell proliferation (paradoxical immunological effect). THP could also bind neutrophils and reduce reactive oxygen species generation, chemotaxis and killing of uropathogenic *Escherichia coli* by engaging sialic acid-binding Ig-like lectin-9 (Siglec-9) receptor through *N*-glycan moieties of sialic acid [80]. The important physiological and immunological functions of THP are listed in Table 1. 

### 3.2. Molecular Basis and Significance of Paradoxical Immunological Effect of THP on Activated Immune Cells

The paradoxical immunological effect of THP may result from the binding of this protein with cytokines in the surrounding milieu and the dynamic production of pro- and anti-inflammatory cytokines by THP-activated PBMCs, as reported by Su et al. [24]. This paradoxical immunological effect of THP on immune cells renders it immunomodulatory in the urinary system for preventing overwhelming tissue damage after microbial invasion. In the vicinity of distal convoluted tubules where acidic pH is present, THP may express its optimal cytokine-binding activity [46]. Nevertheless, the real mechanisms underlying the immunomodulatory effects of THP require further investigation. 

## 4. Molecular Basis of THP-Enhanced PMN Phagocytosis

**Figure 3:**
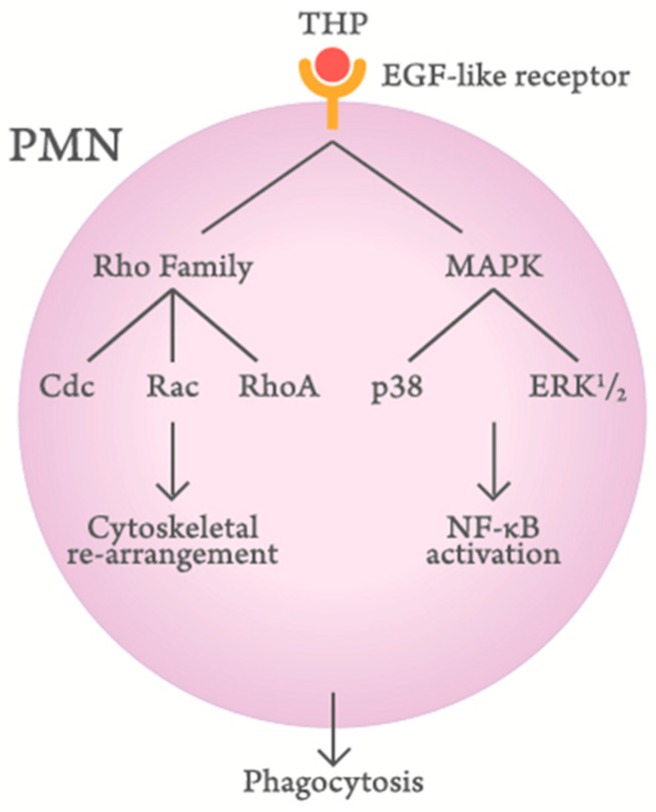
Scheme demonstrating the signaling pathways of Tamm–Horsfall protein (THP)-enhanced polymorphonuclear leukocyte (PMN) phagocytosis.

Siao et al. [79] and Li et al. [36] have investigated the molecular basis of THP-enhanced PMN phagocytosis. They have found that THP, through its EGF-like domains, binds to EGF receptors or surface-expressed lactoferrin and cathepsin G on PMN to activate *Rho* family molecules (Cdc, Rac, and RhoA) and mitogen-activated protein kinase signaling pathways. Finally, the cytoskeletal rearrangement and subsequent nuclear factor-κB phosphorylation enhance PMN phagocytosis. A proposed scheme is demonstrated in Figure 3.

## 5. Pathological Conditions Related to Abnormal THP Expression

### 5.1. Decreased Urinary THP Excretion in Renal Insufficiency may Further Accelerate Renal Inflammation

Investigations have demonstrated decreased urinary THP excretion in endemic nephropathy [81,82], diabetic nephropathy [54,56,70], and lupus nephritis [53]. The decreased urinary THP production is not only a useful biomarker of renal tubular damage [56,57,58] but also an indicator of decreased clearance of systemic and renal proinflammatory cytokines, and it can thus accelerate renal inflammation. The molecular mechanism of this accelerated inflammation is based on the fact that normal THP production can suppress bone marrow granulopoiesis and neutrophil infiltration to prevent damaged kidney-induced inflammation [27]. In addition, decreased THP production in a damaged kidney renders the urinary system susceptible to infection that may further accelerate renal failure.

### 5.2. Aberrant Localization of the THP Molecule Elicits Anti-THP Antibody Production and Interstitial Nephritis

Anti-THP antibody production has been reported in patients with endemic nephropathy [82], recurrent urinary tract infections [83], obstructive uropathy and vesicoureteral reflux [84], medullary cystic disease [28,29], and renal allograft rejection [85]. The abnormal deposition of THP in these diseases results from extensive tubular damage with leakage of THP from its normal intracellular and intraluminal locations into the renal interstitium [86]. Abnormal THP deposition in the renal interstitium attracts and activates PMN and PBMCs and thus elicits inflammatory reactions and anti-THP antibody production. In an animal study, challenging rabbits with homologous urine or purified THP [87] or with an egg white component [88] was observed to elicit anti-THP antibody generation and inflammatory reactions around the TAL of the loop of Henle; this was caused by in situ formation of a THP–anti-THP immune complex [89]. Cainelli et al. [90] demonstrated that a rejected allograft kidney is commonly accompanied by infections and abnormal THP deposition in the renal tubular interstitium to elicit immune responses to THP [90]. Consequently, an in situ THP–anti-THP immune complex is formed in the intercellular space of the TAL, eventually resulting in interstitial nephritis. These results have suggested that the measurement of urinary THP excretion and anti-THP antibodies can predict the onset and prognosis of interstitial nephritis after urinary tract infections [83,84]. 

### 5.3. Implication of UMOD Gene mutations in Urinary Cast/Calculi Nephropathy and Familial Juvenile Hyperuricemic Nephropathy

In vitro experiments have shown that increased concentrations of electrolytes (hypertonic condition), hydrogen ions (low pH), and THP per se would facilitate THP aggregation and subsequent gel formation [41,42]. When such abnormal situations occur in the TAL of the nephron, hyaline casts or even renal calculi are formed [91]. Thus, THP not only protects against calcium oxalate crystal formation [31] but also participates in the pathogenesis of cast nephropathy [92] and urolithiasis [93,94] in some pathological conditions. Liu et al. [32] reported progressive renal capillary calcification and renal stone formation in mice deficient in THP. However, whether THP is a passive or an active participant in stone formation has yet to be determined. Experimental and clinical evidence suggests that tubular obstruction by urinary casts constitutes a major contribution to the development of acute or chronic renal failure. *UMOD* mutations may elicit familial juvenile hyperuricemic nephropathy, glomerulocystic kidney disease, or type 2 autosomal dominant medullary cystic kidney diseases [28,29]. This is because the dysfunctional mutated THP not only potentiates cast/calculus formation but also impairs uric acid and its own excretion through the urinary system [95].

### 5.4. Role of THP in other Pathological Conditions 

A cell-mediated immune response to THP was observed in 90% of patients with primary biliary cirrhosis and/or active/chronic hepatitis associated with renal tubular acidosis [96]. Immunofluorescent staining with rabbit antiserum against human THP demonstrated the presence of THP in the cell membranes of human hepatocytes. This implies that an aberrant immune response to THP is initiated by the release of cross-reactive autoantigens from damaged liver cells that are implicated in autoimmune liver disease associated with secondary renal tubule acidosis.

Previous investigations have also reported an increased aggregation of THP when its overproduction is associated with a Bence–Jones protein [97] or radiocontrast medium-induced nephrotoxicity [98]. In cast nephropathy associated with multiple myeloma, THP was demonstrated in Bowman’s capsule, suggesting that the reflux of urine in a nephron may be caused by retrograde obstruction by a cast [99]. In infants and children with prolonged contrast medium retention after intravenous urography, large amounts of THP are found in urine during the subsequent diuretic stage. Adequate hydration to maintain high urine output and urinary alkalization would theoretically prevent intratubular cast formation. This becomes the therapeutic basis of fluid supplementation for patients with acute renal failure caused by radiocontrast medium. 

## 6. Conclusions and Prospects

THP is a unique urinary mucoprotein with high CHO content synthesized only by renal epithelial cells in the TAL of the loop of Henle. The molecule acts primarily as a defense element for preventing infections and calculus formation in the urinary system. THP mutation, decreased THP production in renal insufficiency, or aberrant THP localization in urinary tract obstruction/vesicoureteral reflux diseases may lead to severe pathological outcomes. An excellent review in these regards has been provided by Devuyst et al. [100]. Therefore, the amount of urinary THP excretion can become a useful biomarker of acute and chronic renal damage. In addition, THP exhibits immunomodulatory effects by activating naïve immune cells and downregulating active immune cells. This renders THP standing on the link of innate and adaptive immunity. This unique paradoxical characteristic of THP might be useful in clinical practice. We anticipate that active motifs or antigenic sites responsible for these intriguing effects of inducing anti-THP antibody will be identified for application in several clinical aspects in the near future. These may include application of the THP TNF-α binding motif as a replacement of monoclonal antibody (MoAb) therapy against TNF-α in the treatment of immune-mediated chronic inflammatory diseases such as rheumatoid arthritis. The strategy may be superior to current MoAb biologics, owing to less immunogenicity and less tachyphylaxis, because the THP fragment is less likely to be recognized by the immune system as “non-self.” Furthermore, the THP IgG-binding motif might be used as a binder of pathogenic autoantibodies in antibody-depleting therapy against catastrophic autoimmune diseases. This may be accomplished by incorporating its IgG-binding motif into hollow fibers as an absorption substrate during plasmapheresis or other forms of renal replacement therapies. Finally, an effective fragment of THP might act as an immune-potentiating motif for immunocompromised patients. This will have a benefit greater than vaccination in that THP bears less risk of causing slow microbial infections that may occasionally be seen with conventional vaccines.

## Figures and Tables

**Table 1:** Physiological and immunological activities of Tamm–Horsfall protein.

Physiological Activities	Immunological Activities
Countercurrent gradient formation in renal tubular ascending limb of Henle’s loop	An immunosuppressive molecule in amniotic fluid to prevent fetal allograft rejection
A 2Cl^−^-K^+^-Na^+^ cotransporter A salt-sensitive molecule to regulate systemic blood pressure A defense molecule against infections in urinary system Prevention of urinary stone formation Increase uric acid excretion Regulator of bone marrow granulopoiesis Suppresion of neutrophil infiltration in damaged kidney Potentiate interstitial mononuclear phagocyte number, plasticity, and phagocytic activity	A renal ligand for systemic cytokine clearance including IL-1β, IL-6, IL-8, TNF-α, and IFN-γ. Binding with serum proteins: - High affinity: human IgG light chain, C1q - Moderate affinity: BSA, cathepsin G - Low affinity: lactoferrin PMN activation in vitro: - Phagocytosis - CR1, CR2 expression - PGE_2_ production
	Immunomodulation on lymphocytes: - Immunopotentiation on naïve cells - Immunosuppression on activated cells - Nonspecific binder for renal glomerular mesangial cell in vitro

BSA, bovine serum albumin; IFN, interferon; IgG, Immunoglobulin G; IL, interleukin; PGE_2_, prostaglandin E_2_; PMN, polymorphonuclear leukocytes; TNF, tumor necrosis factor.
